# Fear of Nurses During COVID-19 Pandemic in Saudi Arabia: A Cross-Sectional Assessment

**DOI:** 10.3389/fpsyg.2021.736103

**Published:** 2021-10-14

**Authors:** Mahaman L. Moussa, Fatchima Laouali Moussa, Homood A. Alharbi, Tagwa Omer, Saleh Abdulkarim Khallaf, Hamad Samran Al Harbi, Ahmed Abdullah Albarqi

**Affiliations:** ^1^College of Nursing, King Saud University, Riyadh, Saudi Arabia; ^2^College of Nursing, Princess Nourah Bint Abdulrahman University, Riyadh, Saudi Arabia; ^3^College of Nursing, King Saud bin Abdulaziz University for Health Sciences, Jeddah, Saudi Arabia; ^4^King Salman Medical City (KSMC), Ministry of Health, Riyadh, Saudi Arabia; ^5^Nursing Administration Hemodialysis Center Hankyah General Hospital, Ministry of Health, Riyadh, Saudi Arabia

**Keywords:** fear, nurses, COVID-19, outbreak, Saudi Arabia

## Abstract

**Objective:** We aimed to assess the level of fear among nurses in Saudi Arabia during the COVID-19 outbreak.

**Methods:** A cross-sectional survey-based study was conducted from June to August 2020. All nurses currently working in public and private hospitals in Saudi Arabia during the COVID-19 pandemic were invited to complete an online survey. We used the 7-item unidimensional Fear of COVID-19 Scale (FCV-19S) to assess the level of fear of COVID-19. Multiple regression analysis was used to identify predictors associated with fear of COVID-19.

**Results:** A total of 969 nurses participated in this study. The participants were relatively young with a mean age of 35.5 ± 10.46 years. About two-thirds of the participants were women (65.9%), married (57.2%), and were non-Saudi nationals (67%). The total mean score for the FCV-19S was 19.7 SD 7.03 (range 7–35), which is near the mid-point, indicating a moderate level of fear of COVID-19. Out of the eight variables measured in the analysis, three variables emerged as a significant predictor (i.e., gender, marital status, and age). A higher level of fear (FCV-19S) was associated with being a woman, married, and older age (*p* ≤ 0.05).

**Conclusion:** This study demonstrated the level of fear of COVID-19 among nurses in Saudi Arabia. Overall, nurses in Saudi reported moderate levels of fear of COVID-19. Assessing the level of fear of nurses who work during the COVID-19 pandemic should be a priority to health care administrators to prevent mental health difficulties or psychological injury.

## Introduction

The coronavirus disease 2019 (COVID-19) outbreak has caused a significant burden globally. Several countries that first faced the COVID-19 shed light on the effects of the pandemic on the health care system and health care providers (Alfieri et al., 2020; Armocida et al., 2020; Oliva et al., 2020). Psychological distress, burnout, and psychosomatic symptoms were reported by health care providers especially those physicians, nurses, and other health care providers that are at the forefront of defense against COVID-19 (Barello et al., 2020; Giusti et al., 2020; Marton et al., 2020). The increasing number of patients with COVID-19 had posed a great impact on health care providers particularly nurses who comprise the largest group of health professionals (Fernandez et al., 2020; Goh et al., 2021). Nurses have multiple roles in this outbreak such as dealing with suspected patients, triaging patients, detecting suspected cases with infections, and providing essential treatment to patients with COVID-19 (Baskin and Bartlett, 2021). Moreover, the long working hours and extra-shift to meet the patients with COVID-19 and the unique needs of the family pose a huge health risk to nurses (Nie et al., 2020). Nurses who are directly involved in treating patients with COVID-19 work under great pressure and stress.

Nurses must face this critical situation and unfav conditions that increase their risk of negative consequences such as psychological distress. The challenges experienced by the nurses in this crisis might not only affect them but also compromise their work and the quality of providing care to their patients (Penwell-Waines et al., 2018). The multiple roles played by the nurses are crucial in this fight against the COVID-19 pandemic. In this sense, preserving the health and well-being of nurses during the COVID-19 pandemic is a significant challenge both in the hospital administrators and policy-makers.

Previous studies showed that the psychological health of nurses is significantly associated with their work performance (Fronteira and Ferrinho, 2011; Oyama and Fukahori, 2015). Psychological distress including fear and anxiety has been reported among health care workers during the COVID-19 outbreak (Amin, 2020; Du et al., 2020). This psychological burden of fear, anxiety, and burnout is likely to result in poor clinical decisions and poor clinical outcomes. The high occurrence of medical errors has been linked to nurses impair cognitive functioning and clinical decision which can put patients at great risk (Zhu et al., 2020). Additionally, acute stress can lead to resignation thoughts that may aggravate shortages of nurses. To alleviate the negative outcome, health authorities must assess the psychological pressure of the impact of the COVID-19 pandemic on nurses.

To effectively provide high-quality care and play their role during an outbreak, nurses need to maintain their psychological and mental health. The psychological effect of the COVID-19 outbreak on nurses should be assessed and monitored. Understanding and assessing these effects are essential to the protection of well-being and emotional resilience of nurses, which directly affect the quality of health care services. To date, there is still a scarcity of epidemiological data on the psychological health of nurses and its associated factors. Thus, the main purpose of this study was to assess the level of fear among nurses in Saudi Arabia during the COVID-19 outbreak. Therefore, the result of this study would contribute to expanding the knowledge on the impact of an outbreak on nurses and at a time of heightened need. This will also assist hospital administrators in developing future workforce policy and institutional response to other waves of this pandemic.

## Methods

### Design and Participants

A cross-sectional survey-based study was conducted from June to August 2020. Due to the current situation and restrictions imposed by the Ministry of Health in Saudi Arabia, convenience sampling was used in this study. A questionnaire link was sent to different nurses through social media platforms (e.g., WhatsApp and Gmail). All nurses currently working in public and private hospitals in Riyadh and Madinah, Saudi Arabia were invited to participate in this study. Ethical clearance was obtained from the Institutional Review Board of King Saud University before data gathering.

### Instruments

All participants answered a two-part questionnaire that assesses the fear about COVID-19. The first part of the questionnaire was demographic characteristics that include age, gender, marital status, monthly income, years of experience in the organization, and years of experience in their profession. The second part of the questionnaire was the Fear of COVID-19 Scale (FCV-19S; Ahorsu et al., 2020). This 7-item unidimensional scale was answered by nurses using a 5-point Likert scale which ranged from 1 (strongly disagree) to 5 (strongly agree). The lowest possible score for the entire scale is seven, and the highest possible score is 35, with a higher score indicating greater fear of COVID-19. Previous research reported excellent predictive validity and reliability (α = 0.86) of the scale while the Cronbach's α of the scale in this study was 0.74 (Ahorsu et al., 2020; Gritsenko et al., 2020).

### Statistical Analysis

We first examined the demographic characteristics of the participants using frequency distribution. All continuous data were presented as mean ± SD while categorical data were presented as frequencies and percentages. The Shapiro-Wilk test was applied to check the normality or distribution of data. Pairwise deletion of cases was used with missing data. Independent *t*-test and one-way ANOVA were used to examine the association between fear of COVID-19 and the demographic characteristics of nurses. Multiple Regression analysis was performed to examine the predictors associated with fear of COVID-19. The dependent variable used is the FCV-19S and the independent variables are the demographic characteristics of the participants (e.g., age, gender, marital status, and nationality). *P*-value was set at <0.05 and considered statistically significant. All data were entered and analyzed using SPSS windows v. 23 (Chicago, Illinois, USA).

## Results

A total of 969 nurses participated in this study. The participants were relatively young with a mean age of 35.5 ± 10.46 years. About two-thirds of the participants were women (65.9%), married (57.2%), and were non-Saudi nationals (67%). Nearly half of the respondents had a salary of less than SR10,000 (1 US$ = SR 3.75). The proportion of the nurses who had been in the organization in <5 years was 25.1%, while 31.8% had been in the organization for more than 10 years. Of note, 30% of the participants had been practicing their profession between 5 and 10 years (*n* = 373, 38.5%). The complete demographic characteristics of the participants are presented in Table 1.

**Table 1:** Demographics of the health care providers.

**Variable**	**Count (*N* = 969)**	**%**
**Age**	Mean 35.5 SD 10.46	
20 – 29	357	36.8
30 – 39	316	32.6
40 and above	296	30.6
**Gender**		
Male	330	34.1
Female	639	65.9
**Marital status**		
Single	415	42.8
Married	554	57.2
**Nationality**		
Saudi	320	33
Non –saudi	649	67
**Hospital type**		
Public	751	77.5
Private	218	22.5
**Monthly income**		
<10,000 SR	298	49
10,000 or more	266	51
**Years of experience in organization**		
<5 years	253	26.1
5–10 years	408	42.1
10 years or more	308	31.8
**Years of experience in practicing profession**		
<5 years	236	24.4
5–10 years	373	38.4
10 years or more	360	37.2

The total mean score for the FCV-19S was 19.7 SD 7.03 (range 7–35) which is near the mid-point that represents that the nurses had a moderate level of fear of COVID-19. The analysis also found that female nurses had a higher mean scale score on FCV-19S than those male nurses (*t* = 0.686, *p* = 0.043). Detailed scores on the FCV-19S by the characteristics of participants are shown in Table 2.

**Table 2:** Characteristics and scores on the fear of COVID-19 scale (FCV-19S) of participants.

**Variable**	**Count (*N* = 969)**	**Statistical test**	***p*-value**
**Fear COVID-19 (FCV-19S)**	Mean 19.7 SD 7.03		
**Age^b^**			
20 – 29	19.7 (6.95)	*F* = 0.497	0.608
30 – 39	19.5 (7.18)		
40 and above	20.0 (6.95)		
**Gender^a^**		*t* = −0.686	**0.043**
Male	19.2 (6.85)		
Female	20.1 (7.13)		
**Marital status^a^**		*t* = 0.676	0.498
Single	19.5 (7.22)		
Married	19.8 (6.89)		
**Nationality^a^**			
Saudi	19.5 (7.29)	*t* = 0.686	0.493
Non – Saudi	19.8 (7.29)		
**Hospital type^a^**		*t* = 0.721	0.262
Public	19.7 (7.26)		
Private	19.0 (7.18)		
**Monthly income^a^**		*t* = −0.975	0.330
<10,000 SR	19.5 (7.06)		
10,000 or more	19.9 (7.01)		
**Years of experience in organization^b^**		*F* = 1.90	0.150
<5 years	19.0 (7.26)		
5–10 years	20.1 (6.82)		
10 years or more	19.8 (7.11)		
**Years of experience in practicing profession^b^**			
<5 years	19.4 (7.18)	*F* = 0.517	0.596
5–10 years	19.7 (6.83)		
10 years or more	20.0 (7.07)		

a *t-test for the independent group*.

b *ANOVA. Bold values indicates significant at 0.05*.

The total score is the sum of the scores of the seven items, ranging from 7 to 35, with a higher score indicating greater fear of COVID-19.

Out of the eight variables measured in the analysis (i.e., age, gender, marital status, nationality, monthly income, years of experience in organization, and years of experience practicing their profession), three variables (i.e., gender, marital status, and age) emerged as a significant predictor. Higher level of fear (FCV-19S) was associated with being woman (beta = 0.34, 95% CI −0.60 to 1.29), married (beta = 0.17, 95% CI −0.81 to 1.15), and older age (beta = 0.01, 95% CI −0.04 to 0.08). No significant association was found between FCV-19S and nationality, monthly income, years of experience in organization, and years of experience practicing their profession. We used multiple regression analysis to assess the predictors associated with fear of COVID-19 (Table 3).

**Table 3:** Predictors of the fear of COVID-19.

**Variable**	**B**	**SE**	**95% CI**	***p*-value**
Age	0.01	0.03	−0.04 – 0.08	**0.017**
Gender	0.34	0.48	−0.60 – 1.29	**0.035**
Marital Status	0.17	0.50	−0.81 – 1.15	**0.048**
Nationality	−0.51	0.52	−1.54 – 0.52	0.733
Hospital type	0.55	0.46	−0.32 – 1.82	0.383
Monthly income	0.17	0.57	−0.96 – 1.30	0.331
Years of experience in organization	0.69	0.75	−0.78 – 2.18	0.767
Years of experience in practicing profession	−0.54	0.78	−2.08 – 1.00	0.357

*CI, confidence interval; SE, standard error; B, standardized regression coefficient*.

*p-value significant at p < 0.05. Bold values indicates significant at 0.05*.

## Discussion

The COVID-19 pandemic has caused a huge burden and serious concern among health care providers. The outbreak brought an unprecedented impact on nurses such as adverse psychological problems (e.g., fear). The present results of this study revealed that nurses in Saudi Arabia had a moderate level of fear of COVID-19. Our results also showed that age, gender, and marital status of nurses are significant predictors of fear of the COVID-19 pandemic.

The level of fear of COVID-19 found in this study is comparable with the results of previous studies conducted in the Philippines (*M* = 19.6 ± 6.12), Jordan (*M* = 23.6 ± 10.8), and Vietnam (*M* = 16.7 ± 5.3) (Nguyen et al., 2020; Alnazly et al., 2021; Labrague and de Los Santos, 2021). This implies that nurses had a moderate level of fear months after the pandemic. Several years ago, nurses in Saudi Arabia encountered and combatted multiple infectious disease outbreaks such as severe acute respiratory syndrome (SARS) and the Middle East respiratory syndrome-related coronavirus (MERS-COV). The emergence of these infectious disease outbreaks caused emotional distress among nurses in Saudi Arabia (Bukhari et al., 2016; Khalid et al., 2016). The level of fear expressed by the nurses in this study may be due to the severity of COVID-19, risk of infection, and fear of the unknown, which was similarly reported in nurses in other countries (Nguyen et al., 2020; Nie et al., 2020; Labrague and de Los Santos, 2021). These factors intensified the level of fear and heightened emotions of nurses in Saudi Arabia that may lead to psychological and emotional disorders.

Another highlight of this study is the factors associated with the fear of nurses for COVID-19. This study found that female nurses reported higher fear scores than male nurses. This is in accordance with the findings among medical students in Vietnam (Nguyen et al., 2020). However, this is contrary to the findings of a recent study among Jordanian nurses in which fear mean scores were greater among men than in women (Alnazly et al., 2021). The higher level of fear among female nurses maybe because they are usually posed or suffer stressful life events such as house works and caregiver roles than male nurses (Harkness et al., 2010; Nguyen et al., 2020). Hence, a possible reason for this inconsistency was based on the psychological response and coping mechanism of nurses. Fear is associated with the desire of individuals to evade and protect themselves from certain situations (Fredrickson, 2001; Huang et al., 2020). Those who reported more fear have active-oriented coping strategies than those who had less fear of avoiding certain problems (Huang et al., 2020). Thus, a coping strategy program may help nurses in managing these stressful life events such as the COVID-19 pandemic.

This study also showed that age, gender, and marital status of nurses are significant predictors of fear of the COVID-19 pandemic. Our results are in line with a recent study conducted in Saudi Arabia in which the age of physicians was related to the increased fear and worry of COVID-19 (Al Sulais et al., 2020). Furthermore, the results are consistent with a previous study conducted in China that female nurses were more likely to have high levels of fear than male nurses (Huang et al., 2020). Meanwhile, previous reports showed that nurses who were married and with children had higher levels of fear than single, widowed, and separated nurses (Fu et al., 2021). A possible reason for this fear among married nurses was the fear of carrying the virus from their work and transferring it to their families. These findings may have contributed to the emotional burden of nurses at the time of the pandemic.

The fear experienced by the nurses may be due to different factors such as the rapid surge of the COVID-19 cases and deaths in Saudi Arabia and other countries (Mekonen et al., 2021). Another reason is that nurses are considered frontliners who are directly involved in treating patients with COVID-19, and this could contribute to their feelings of fear of being infected and fear of infecting others or family members. Finally, fear of the unknown, i.e., in the early phase of the COVID-19 outbreak, there is no specific medication and knowledge of the mode of transmission of the disease. These factors made nurses at risk of having psychological and emotional problems. Several strategies were recommended to reduce the fear, anxiety, and emotional burden of nurses during the COVID-19 outbreak. The WHO advised health care workers to engage in physical activities, eat healthy food and have sufficient rest to mitigate the negative psychological impact of COVID-19. To reduce the feeling of isolation, health care workers are encouraged to used social platforms to get connected with their families, friends, and colleagues^1^ (Naeim et al., 2020). To support and protect health care workers including nurses, social support is necessary as a coping mechanism to decrease the emotional impact of COVID-19. Health care administrators and healthcare leaders should rapidly implement policy changes to improve the well-being of nurses.

A few limitations of this study can be noted. First, the design of this study, in which cross-sectional study design cannot determine causality. Then, this study was conducted as self-administered, which may result to be biased. Finally, although the sample of this study was large, it is only conducted in two provinces in Saudi Arabia, which makes it difficult to generalize the results to the entire population of nurses in Saudi Arabia. However, this study may form preliminary data for future researchers for comparison.

## Conclusion

We found moderate levels of fear of COVID-19 among nurses in Saudi Arabia. This study also identified significant predictors of fear of COVID-19 among nurses. Factors determined to be associated with the level of fear of COVID-19 among nurses were being women, married, and older age. Assessing the level of fear of nurses who work during the COVID-19 pandemic should be a priority to health care administrators to prevent mental health difficulties or psychological injury. We highlighted vital factors that may help health care administrators in formulating strategic public health interventions in reducing the fear experienced by the nurses in Saudi Arabia. Future studies should focus on exploring interventions to improve nurses coping strategies to the different unprecedented situations such as the COVID-19 pandemic.

## Data Availability Statement

The data set used is locked and stored at College of Nursing, Medical Surgical Department, Princess Nourah Bint Abdulrahaman University and can be obtained from the author on reasonable request.

## Ethics Statement

The studies involving human participants were reviewed and approved by Institutional Review Board of King Saud University. The patients/participants provided their written informed consent to participate in this study.

## Author Contributions

All authors contributed to data analysis, interpretation of results, writing the manuscript, and agreed to be accountable for all aspects of this study.

## Conflict of Interest

The authors declare that the research was conducted in the absence of any commercial or financial relationships that could be construed as a potential conflict of interest.

## Publisher's Note

All claims expressed in this article are solely those of the authors and do not necessarily represent those of their affiliated organizations, or those of the publisher, the editors and the reviewers. Any product that may be evaluated in this article, or claim that may be made by its manufacturer, is not guaranteed or endorsed by the publisher.

## Correction note

This article has been corrected with minor changes. These changes do not impact the scientific content of the article.

## Footnotes

^1^World Health Organization (2020). Available from: https://www.who.int/docs/default-source/coronaviruse/mental-health-considerations.pdf [Last accessed on 2021 Apr 10].
